# From autoimmune inflammation to malignancy: causal genetic evidence for the RA-squamous cell lung cancer axis

**DOI:** 10.1186/s13075-025-03703-8

**Published:** 2025-12-01

**Authors:** Jianbin Li, Suiran Li, Yuxiu Ka, Siwei Wang, Gesang Yuzhen, Wei Liu

**Affiliations:** 1 Department of Rheumatism and Immunity, First Teaching Hospital of Tianjin University of Traditional Chinese Medicine, Tianjin, People’s Republic of China; 2 National Clinical Research Center for Chinese Medicine Acupuncture and Moxibustion, Tianjin, People’s Republic of China

**Keywords:** Rheumatoid Arthritis, Squamous Cell Lung Cancer, Propensity Score Matching, Mendelian Randomization, Causal Inference, Evidence Triangulation

## Abstract

**Background:**

The association between rheumatoid arthritis (RA) and lung cancer risk has attracted considerable clinical attention, but conclusions remain inconsistent due to confounding factors such as smoking, and previous studies have paid less attention to specific histological subtypes. This study aims to systematically elucidate the complex relationship between the two through an evidence triangulation strategy combining large-scale clinical cohort studies, machine learning risk prediction, and genetic causal inference.

**Methods:**

This study adopted an evidence triangulation strategy, integrating evidence from different populations and methods. First layer of evidence (clinical association, Chinese population): We conducted 1:1 propensity score matching analysis on a real-world cohort of 8,867 subjects (4,661 RA patients) (2014–2024, First Teaching Hospital of Tianjin University of Traditional Chinese Medicine), with particular focus on the association between RA and different lung cancer subtypes (adenocarcinoma, squamous cell carcinoma, small cell carcinoma). We also built machine learning models (logistic regression, random forest, XGBoost) based on 22 clinical features from this cohort to explore the feasibility of individualized risk prediction for overall lung cancer. Second layer of evidence (genetic causation, European population): We turned to use completely independent large-scale public GWAS summary data (RA: *N* = 92,044, European ancestry; squamous cell lung cancer: *N* = 63,053, European ancestry; smoking: *N* = 111,752, European ancestry), employing multivariable Mendelian randomization (MVMR) analysis, after adjusting for the genetic effects of smoking, to specifically explore the causal effect of RA on squamous cell lung cancer.

**Results:**

Clinical evidence reveals that the association between RA and lung cancer exhibits significant subtype specificity: after matching, RA significantly increases squamous cell lung cancer risk (adjusted OR = 2.415, 95% CI: 1.104–5.283, *P* = 0.027), but shows no significant association with adenocarcinoma (*P* = 0.437) or small cell carcinoma (*P* = 0.564). Machine learning models based on clinical features showed limited predictive ability (AUC = 0.57–0.68), revealing the challenge of translating population associations into individual predictions. In an independent European population, genetic analysis presents an instructive paradox: preliminary univariable MR analysis shows RA has a genetic protective effect on squamous cell lung cancer (OR = 0.985), while linkage disequilibrium score regression (LDSC) shows no significant genetic correlation between the two (rg = -0.019, *P* = 0.806). After simultaneously adjusting for the genetic effects of smoking in multivariable MR analysis, the causal promoting effect of RA on squamous cell lung cancer was revealed (OR = 1.02, 95% CI: 1.00–1.04, *P* = 0.046), explaining that the apparent protective effect observed in univariable analysis was actually caused by smoking confounding.

**Conclusion:**

RA specifically increases squamous cell lung cancer (but not other subtypes) risk. Multivariable Mendelian randomization resolves the causal paradox caused by smoking confounding. Clinical evidence (China) and genetic evidence (Europe) come from different populations and require further validation. Results support implementation of targeted squamous cell lung cancer screening for high-risk RA patients.

**Supplementary Information:**

The online version contains supplementary material available at 10.1186/s13075-025-03703-8.

## Introduction

Rheumatoid arthritis (RA) is a chronic autoimmune inflammatory disease affecting approximately 0.5–1% of the global population, characterized by persistent synovitis and systemic inflammation. The inflammation is not limited to joint destruction but involves various extra-articular manifestations. RA patients face an increased burden of comorbidities, including cardiovascular diseases, osteoporosis, and infections, with the potential association with certain malignancies attracting particular attention [1]. The relationship between RA and lung cancer risk is especially concerning, as lung cancer is the leading cause of cancer-related deaths globally and occurs with higher frequency in the RA patient population [2]. However, previous studies have mostly treated lung cancer as a single endpoint, paying less attention to potentially different associations among different histological subtypes (such as adenocarcinoma and squamous cell carcinoma).

The relationship between RA and lung cancer has been highly controversial in medical literature, with numerous methodological challenges making it difficult to establish a clear relationship. Multiple observational studies and meta-analyses have indicated that RA patients have a 40–60% increased lung cancer risk compared to the general population [2–4]. However, smoking, as the most important confounding factor in this relationship, is both a major risk factor for lung cancer and a known environmental trigger for RA development, especially in genetically susceptible individuals carrying HLA-DRB1 shared epitope alleles [5, 6]. Traditional observational studies struggle to completely disentangle the effects of smoking from RA risk itself, as smoking data is often incompletely collected and subject to recall bias [7]. Other possible confounders include occupational exposure, air pollution, and immunosuppressive drugs used in RA treatment, which may increase cancer risk by suppressing immune surveillance or reduce cancer risk by controlling inflammation [4]. Artificial intelligence and machine learning technologies offer new possibilities for disease prediction and risk stratification in clinical medicine. These advanced computational methods have shown potential in identifying complex patterns in high-dimensional clinical data that may not be apparent through traditional statistical methods [8]. However, the performance of machine learning models in predicting rare and heterogeneous events (such as cancer development) in specific patient populations remains uncertain [9]. Accurate prediction of individual lung cancer risk in RA patients using readily accessible clinical parameters would represent a major clinical advance, potentially driving targeted screening strategies and early intervention. However, the inherent complexity of cancer development, involving multifactorial interactions of genetic susceptibility, environmental exposure, and stochastic events, presents enormous challenges for predictive modeling.

Therefore, although clinical observations suggest an association between RA and lung cancer, the causal relationship and underlying genetic mechanisms remain unclear due to the presence of strong confounding factors. This is the core problem our study aims to address. To overcome the inherent limitations of observational studies and explore causality from a genetic perspective, Mendelian randomization (MR) serves as a powerful tool for causal inference, using genetic variants as instrumental variables to study causal relationships while minimizing confounding and reverse causation. To comprehensively explore this complex relationship, this study adopted an innovative multi-dimensional analytical strategy. We combined large-scale propensity score matching cohort analysis (in Chinese population), machine learning predictive modeling, and genetic causal inference (in European population) to explore the association between RA and squamous cell lung cancer from different perspectives and in different populations. This evidence triangulation strategy is a recommended method in modern epidemiology [10, 11], which can provide more robust causal inference than single studies by integrating evidence from different data sources using different methodologies, while revealing consistency and differences in associations across different populations and study designs. We also explored potential shared biological mechanisms through exploratory analysis of common causal genes, but it should be emphasized that these findings should be regarded as hypothesis-generating in nature.

## Methods

### Real-world study design and subjects

This study employed a retrospective cohort study design aimed at investigating the association between rheumatoid arthritis (RA) and squamous cell lung cancer. Study data were derived from the electronic medical record system of the First Teaching Hospital of Tianjin University of Traditional Chinese Medicine, covering RA patients treated between 2014 and 2024. The study group included 4,661 RA patients, with a control group of 4,111 non-RA patients, all treated at the same hospital during the same period. Inclusion criteria included: (1) RA group: patients clinically diagnosed with RA by rheumatology specialists who met the American College of Rheumatology (ACR) and European League Against Rheumatism (EULAR) 2010 RA classification criteria [12] (Note: these are classification criteria rather than diagnostic criteria, mainly used for patient inclusion in research, with high specificity), with complete clinical and laboratory records; (2) Control group: non-RA patients treated during the same period without a history of rheumatic immune diseases. Exclusion criteria were: pre-existing lung cancer history at enrollment, lack of clear diagnostic basis, missing key clinical data, and loss to follow-up during the observation period. The primary exposure factor was RA clinically diagnosed by rheumatology specialists. The primary outcome was newly developed lung cancer and its histological subtypes (adenocarcinoma, squamous cell carcinoma, small cell carcinoma, etc.) during the observation period. Lung cancer diagnosis was based on histopathological examination as the gold standard. For patients unable to obtain pathological specimens for medical reasons (< 5%), comprehensive clinical-imaging diagnosis was used: typical malignant tumor CT/PET-CT imaging features, combined with elevated tumor markers and clinical manifestations, confirmed by multidisciplinary team consultation. All diagnoses were reviewed by at least two independent senior oncologists. We collected diagnosis dates and clinical staging information for all lung cancer patients and recorded their pathological types in detail (such as adenocarcinoma, squamous cell carcinoma, small cell carcinoma, and other types). Collected data included: demographic characteristics (age, sex), comorbidities (hypertension, diabetes, hyperlipidemia), laboratory indicators (ESR, CRP), RA disease characteristics (disease duration), and treatment status (glucocorticoids, immunosuppressants, small molecule targeted drugs, etc.). To control for confounding factors, propensity score matching (PSM) method was used. Propensity scores were constructed based on sex, age, hypertension, diabetes, and hyperlipidemia, using nearest neighbor matching algorithm (caliper = 0.2) for 1:1 matching, ultimately obtaining 1,552 matched pairs. Matching quality was assessed using standardized mean differences (SMD), with SMD < 0.1 considered as good balance. After matching, age and hypertension had SMD > 0.1, which were adjusted as covariates in subsequent logistic regression. Based on matched samples, conditional logistic regression was used to analyze the association between RA and different lung cancer subtypes, with results presented as odds ratios (OR) and 95% confidence intervals (CI). Sensitivity analyses included comparison of different matching algorithms (nearest neighbor, genetic matching) and matching ratios (1:1, 1:2). Restricted cubic spline models were used to analyze nonlinear relationships between age, inflammatory indicators, and squamous cell lung cancer risk. This study was approved by the Ethics Committee of the First Teaching Hospital of Tianjin University of Traditional Chinese Medicine (TYLL2025[Z]005).Due to the retrospective nature of our clinical cohort study, comprehensive smoking data were not systematically available in the electronic medical records. Therefore, smoking could not be included as a covariate in the propensity score matching analysis. This limitation was specifically addressed through the multivariable Mendelian randomization analysis using independent GWAS data (described below), which allowed us to adjust for the genetic effects of smoking when examining the causal relationship between RA and squamous cell lung cancer.

### Construction and evaluation of risk prediction models

Given that clinical analysis revealed a specific association between RA and squamous cell lung cancer, we constructed machine learning models (logistic regression, random forest, XGBoost) based on 22 clinical features to explore the feasibility of individualized risk prediction. The dataset was divided into training and test sets at an 80:20 ratio, with bagged tree imputation used to handle missing values. Model training employed tenfold cross-validation and upsampling techniques to address class imbalance, with AUC as the optimization metric. Model performance was evaluated through test set AUC, calibration curves, and decision curve analysis, with SHAP values applied to quantify the contribution of each feature to prediction results. All analyses were completed in R language environment using caret, xgboost, and dcurves packages, with random seed (123) set to ensure reproducibility.

### Mendelian randomization study design

**Fig. 1:**
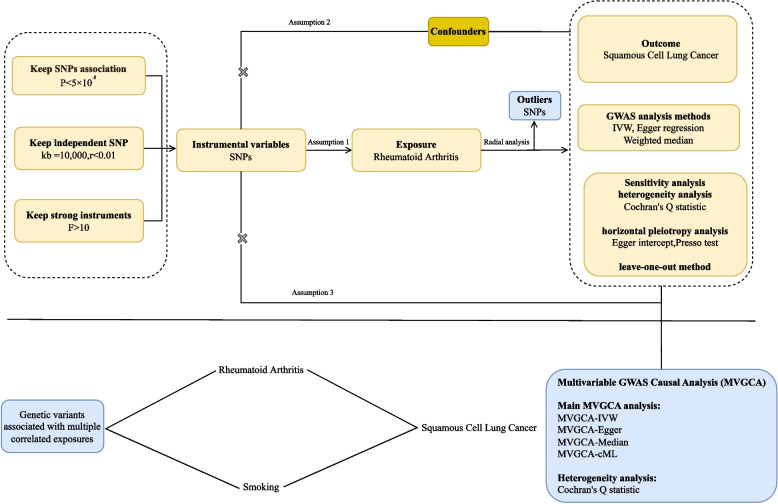
Study Design and Statistical Analysis Flowchart. This figure depicts the complete technical pathway from data source screening, instrumental variable (SNP) extraction to final statistical analysis in this study. Mendelian randomization analysis strictly follows its three core assumptions: (**a**) Relevance: instrumental variables are strongly correlated with exposure factors; (**b**) Independence: instrumental variables are independent of any confounders; (**c**) Exclusivity: instrumental variables can only affect outcomes through exposure factors. All statistical analyses were executed in R language environment (version 4.3.1), with core analyses relying on TwoSampleMR (version 0.5.11) and RadialMR (version 1.1) packages to implement Mendelian randomization analysis and conduct comprehensive sensitivity testing (including heterogeneity, pleiotropy assessment, and multivariable analysis). This flowchart was created using Adobe Illustrator (2024 version)

This study is based on public GWAS summary data for genetic causal inference. It should be noted that this part of the analysis uses GWAS data from European ancestry populations, completely independent from our clinical cohort (Chinese population). Instrumental variable (IV) screening criteria: 1) strongly associated with RA (*P* < 5 × 10⁻⁸, LD r^2^ < 0.001, distance > 10,000 kb); 2) independent of confounders; 3) excluding pleiotropic variants. All IVs had F-statistics > 10, excluding weak instrument bias. Causal effect estimation employed inverse variance weighted (IVW) method as the primary approach, supplemented by Egger regression and weighted median method for sensitivity verification. Bias was controlled through the following strategies: 1) Cochran’s Q test to assess heterogeneity; 2) Egger intercept test to identify horizontal pleiotropy; 3) leave-one-out analysis to exclude single SNP-driven effects; 4) Radial IVW to identify outlier genetic variants. To control for smoking confounding, multivariable Mendelian randomization (MVMR) analysis was performed, simultaneously incorporating genetic effects of RA and smoking. MVMR employed three methods: multivariable IVW, multivariable Egger, and Median method [13–15]. The final analysis included 2,198 valid SNPs. The study design flowchart is shown in Fig. 1.

### Data source and instrumental variables selection

Rheumatoid arthritis data came from GWAS Catalog (https://www.ebi.ac.uk/gwas/), including phenotype, genotype, and clinical information from 92,044 individuals of European ancestry; squamous cell lung cancer data came from GWAS Catalog (https://www.ebi.ac.uk/gwas/), including phenotype, genotype, and clinical information from 63,053 individuals of European ancestry; smoking data came from GWAS Catalog (https://www.ebi.ac.uk/gwas/), including phenotype, genotype, and clinical information from 111,752 individuals of European ancestry, with detailed information in Supplementary Table 1. Although the above GWAS data come from different studies, since many large genetic studies (such as UK Biobank, FinnGen, etc.) are widely used for GWAS analysis of different phenotypes, we cannot completely rule out the possibility of partial sample overlap between these European population cohorts. Sample overlap mainly affects standard error estimation, possibly leading to slightly narrower confidence intervals, but typically does not systematically bias the direction or magnitude of causal effect estimates. It is worth noting that the linkage disequilibrium score regression (LDSC) analysis method we used is robust to sample overlap, so genetic correlation estimates are not affected by this. Instrumental variable (IV) screening in this study was based on the following criteria: genome-wide significance threshold was set (*p* < 5 × 10⁻⁸), and LD threshold (LD r^2^ < 0.001, kb = 10,000) was adopted to ensure effective linkage disequilibrium (LD) [16]. To eliminate weak instrument bias, we calculated F-statistics for each SNP. Instrumental variables were screened through F-statistic method: F-statistic = R^2^ × (N − 2)/(1 − R^2^), where R^2^ = 2 × EAF × (1 − EAF) × Beta^2^ [17, 18]. All SNPs with F-statistics < 10 were considered weak instruments and excluded from further analysis. All instrumental variables included in our final analysis had F-statistics > 10 (see Supplementary Table 2), far exceeding the weak instrument threshold, ensuring the reliability of causal inference. Meanwhile, considering possible differences between proxy SNPs and original SNPs, we did not include proxy SNPs [19, 20]. Finally, Radial MR method was used to remove outliers, further ensuring the reliability of causal inference [21].

### Linkage disequilibrium score regression (LDSC) analysis

LDSC was used to estimate genetic correlation between RA and squamous cell lung cancer. LDSC is based on linkage disequilibrium principles, inferring genetic sharing between traits by calculating LD scores of SNPs, unaffected by sample overlap. Analysis was completed in R language using ldscr package, calling the built-in 1000 Genomes European population LD reference panel (ancestry = 'EUR').

### Identification of shared causal genes

After completing the main Mendelian randomization analysis, we conducted an independent exploratory analysis to identify potential shared causal genes. It is important to note that this module differs significantly from the main MR analysis in analytical strategy and statistical thresholds: The main MR analysis used standard GWAS significance thresholds (*P* < 5 × 10⁻⁸) to screen instrumental variables, assessing the overall causal effect of RA on squamous cell lung cancer, without pre-excluding genetic variants associated with both diseases. This design follows standard Mendelian randomization practice, ensuring assessment of RA's complete genetic effect. Shared gene identification uses a completely different approach: utilizing eQTL data to map disease-associated SNPs to gene expression levels, conducting single-gene MR analysis separately for RA and squamous cell lung cancer. Due to the statistical power limitations of single-gene MR, we used a more lenient threshold (*P* < 0.05) to identify genes with potential causal effects on each disease, then obtained shared causal genes by intersection. The shared gene identification analysis has two compounded methodological weaknesses: (1) single-gene MR has lower power compared to genome-wide MR, and the P < 0.05 threshold leads to a higher false-positive rate; (2) the extremely limited number of genes identified (N = 7) is insufficient to support robust statistical inference. Therefore, all findings from this module should be regarded as highly exploratory, hypothesis-generating in nature, requiring larger-scale genetic studies and independent experimental validation. These shared genes may represent new genetic signals independent of known susceptibility loci identified by traditional GWAS, but this interpretation needs to be treated with caution.

### Functional enrichment and protein–protein interaction (PPI) network analysis

Due to the limited number of shared genes (*N* = 7), functional enrichment analysis has insufficient statistical power and serves only as descriptive exploration. ClusterProfiler was used for GO and KEGG pathway analysis, with *P* < 0.05 considered significant. STRING database (v11.5) was used to construct protein–protein interaction (PPI) network, with Cytoscape (v3.9.1) for visualization.

### Statistical analyses

**Fig. 2:**
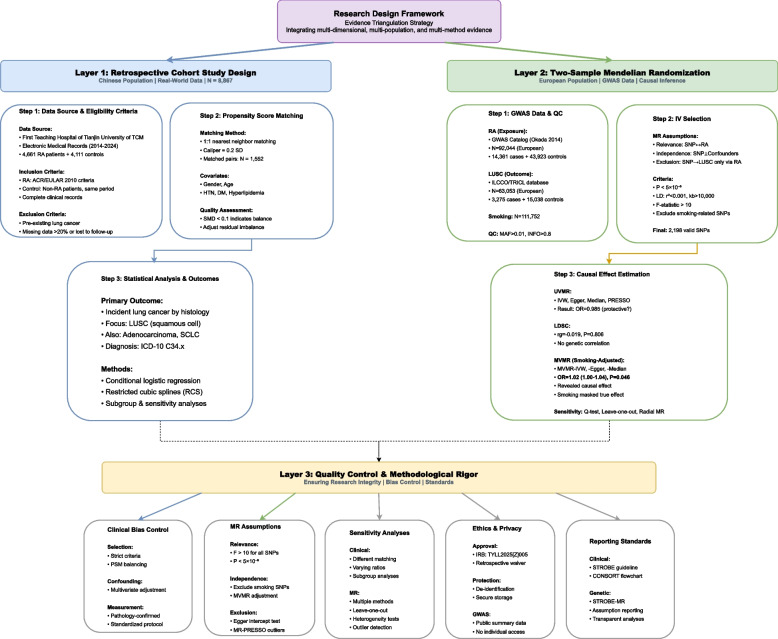
Evidence Triangulation Study Flowchart for RA and Squamous Cell Lung Cancer Association

In real-world data analysis, PSM was used to control confounding (MatchIt package, caliper = 0.2), with unbalanced variables after matching adjusted in multivariable analysis. Restricted cubic splines (RCS) explored nonlinear relationships between continuous variables and risk. Machine learning models employed tenfold cross-validation and upsampling, evaluated through AUC, calibration curves, and decision curve analysis (DCA), with SHAP values for model interpretation. In genetic analysis, IVs were strictly screened (P < 5 × 10⁻⁸, r^2^ < 0.001, F > 10), with IVW and various sensitivity tests (MR-Egger, MR-PRESSO) used for univariable MR (UVMR), LDSC to estimate genetic correlation, and MVMR to adjust for smoking confounding. All analyses employed two-sided tests, with *P* < 0.05 considered significant. Software included R (version 4.2.1, mainly using caret, TwoSampleMR, ldscr packages) and SPSS 25.0. The overall process is shown in Fig. 2.

## Results

### Propensity score distribution before and after matching

**Fig. 3:**
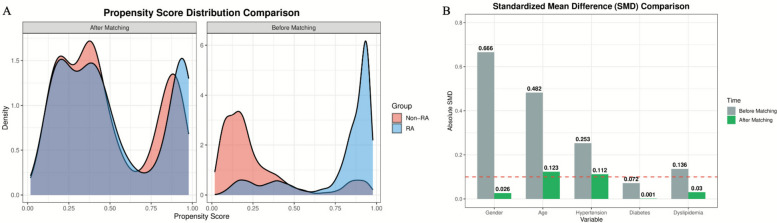
Distribution of Propensity Scores Before and After Matching for RA and Non-RA Patient Groups

To control for the influence of potential confounding factors, this study performed propensity score matching between RA patients and non-RA patients. Figure 3A shows the distribution of propensity scores before matching (right panel) and after matching (left panel) for both patient groups. Before matching, the propensity score distributions of the RA group (blue) and non-RA group (red) were markedly different, indicating significant imbalance in baseline characteristics between the two groups. After 1:1 nearest neighbor matching (caliper = 0.2), with 1,552 matched pairs, the propensity score distribution curves of the samples (left panel) almost completely overlapped, indicating that the overall baseline characteristics of both groups tended to be consistent. Figure 3B shows the SMD of each covariate before and after matching. Before matching (gray bars), the SMDs for sex, age, hypertension, and hyperlipidemia were all far greater than 0.1. After matching (green bars), both groups achieved good balance (SMD < 0.1) in sex (SMD = 0.026), diabetes (SMD = 0.001), and hyperlipidemia (SMD = 0.030). The SMDs for age (SMD = 0.123) and hypertension (SMD = 0.112) were also substantially reduced compared to before matching, approaching the balance threshold of 0.1. For variables that remained imbalanced after matching (i.e., age and hypertension), this study incorporated them into the logistic regression model for further adjustment as covariates in subsequent analyses.

### Baseline characteristics

This study initially enrolled 8,867 subjects, including 4,661 rheumatoid arthritis (RA) patients and 4,111 non-RA controls. Before propensity score matching (PSM), significant differences existed between the two groups in demographic characteristics, comorbidities, and most laboratory indicators (see Table 1 for details). Particularly regarding lung cancer prevalence, the RA group was significantly higher than the non-RA group (1.63% vs. 0.46%, *P* < 0.001). Subtype analysis showed that the RA group had significantly higher prevalence rates of squamous cell carcinoma (0.68% vs. 0.22%, *P* = 0.001), adenocarcinoma (0.62% vs. 0.22%, *P* = 0.002), and other types diagnosed by imaging (0.25% vs. 0%, *P* = 0.001). Table 2 presents baseline characteristics after matching. According to SMD (standardized mean difference) assessment, good balance was achieved between the two groups after matching in sex (SMD = 0.026), diabetes (SMD = 0.001), and hyperlipidemia (SMD = 0.030) (SMD < 0.1). However, the overall lung cancer prevalence in the RA group remained significantly higher than the non-RA group (2.32% vs. 1.22%, *P* = 0.021). More importantly, subtype analysis revealed significant heterogeneity: the overall difference between the two groups after matching was mainly driven by significantly increased prevalence rates of squamous cell carcinoma (1.48% vs. 0.58%, *P* = 0.013) and other types diagnosed by imaging (0.32% vs. 0%, *P* = 0.025). In contrast, the prevalence rates of adenocarcinoma (0.38% vs. 0.58%, *P* = 0.437) and small cell carcinoma (0.13% vs. 0.06%, *P* = 0.564) showed no significant differences between the two groups. This finding suggests that the association between RA and lung cancer has significant subtype specificity, mainly concentrated in squamous cell carcinoma. Additionally, several baseline variables in the matched RA group did not achieve balance (SMD > 0.1), including age (SMD = 0.123), hypertension (SMD = 0.112), inflammatory markers (ESR SMD = 0.318; CRP SMD = 0.120), accompanied by specific lipid (such as TG SMD = 0.277; HDL SMD = 0.352) and glucose (SMD = 0.213) metabolic patterns.

**Table 1:** Comparison of Baseline Characteristics Between RA and Non-RA Patients Before Propensity Score Matching

Character	Total	RA Group	Non-RA Group	*P*-value
Basic Information	8867	4661	4111	-
Gender (Male), n (%)	4659(52.54)	1020(21.88)	3639(88.52)	0.001
Age (years)	60.34 ± 13.29	62.84 ± 11.26	57.48 ± 14.79	0.001
Lung Cancer, n (%)	95(1.07)	76(1.63)	19(0.46)	0.001
squamous cell carcinoma	41(0.46)	32(0.68)	9(0.22)	0.002
adenocarcinoma	38(0.43)	29(0.62)	9(0.22)	0.005
small cell carcinoma	4(0.04)	3(0.06)	1(0.02)	0.387
Others (Radiological Diagnosis)	12(0.13)	12(0.25)	0	0.001
Systolic BP (SBP, mmHg)	139.79 ± 20.13	139.86 ± 20.08	139.71 ± 20.08	0.740
Diastolic BP (DBP, mmHg),	87.83 ± 12.53	87.86 ± 12.51	87.86 ± 12.55	0.768
Hypertension, n (%)	4193(47.29)	1682(36.09)	2511(61.08)	0.001
Diabetes, n (%)	1371(15.46)	574(12.31)	797(19.39)	0.001
Hyperlipidemia, n (%)	1168(13.17)	323(6.93)	845(20.55)	0.001
Fasting Blood Glucose (mmol/L)	5.75 ± 2.01	5.39 ± 1.75	6.15 ± 2.19	0.001
Total Cholesterol (CHO, mmol/L), Mean ± SD	4.54 ± 1.25	4.66 ± 1.27	4.41 ± 1.22	0.001
Triglycerides (TG, mmol/L)	1.73 ± 1.79	1.34 ± 1.61	2.18 ± 1.88	0.001
High-Density Lipoprotein (HDL, mmol/L)	1.07 ± 0.32	1.16 ± 0.34	0.96 ± 0.26	0.001
Low-Density Lipoprotein (LDL, mmol/L)	2.75 ± 0.92	2.86 ± 0.91	2.62 ± 0.92	0.001
Alanine Aminotransferase (ALT,U/L)	23.33 ± 24.63	18.01 ± 17.57	29.31 ± 29.59	0.001
Aspartate Aminotransferase (AST,U/L)	20.92 ± 17.12	19.35 ± 16.29	22.70 ± 17.85	0.001
C-Reactive Protein (CRP,mg/L)	28.13 ± 40.90	27.17 ± 37.39	30.00 ± 46.91	0.010
Erythrocyte Sedimentation Rate (ESR, mm/h)	45.25 ± 32.86	50.17 ± 32.81	35.8 ± 30.86	0.001
RA Duration (years)	12.6 ± 8.89	12.6 ± 8.89	-	-
Rheumatoid Factor (RF,IU/mL)	212.13 ± 524.75	212.13 ± 524.75	-	-
Cyclic Citrullinated Peptide (CCP,IU/mL)	270.21 ± 445.01	270.21 ± 445.01	-	-
Medication Use				
Use of Lipid-Lowering Drugs, n (%)	1457(16.43)	1457(31.26)	-	-
Use of Antidiabetic Drugs, n (%)	968(10.92)	968(20.77)	-	-
Use of Antihypertensive Drugs, n (%)	2124(23.95)	2124(45.57)	-	-
Steroids, n (%)	648(7.31)	648(13.90)	-	-
Immunosuppressants, n (%)	1105(12.46)	1105(23.71)	-	-
Baricitinib, n (%)	28(0.32)	28(0.60)	-	-
Methotrexate (MTX), n (%)	253(2.85)	253(5.43)	-	-
Hydroxychloroquine (HCQ), n (%)	159(1.79)	159(3.41)	-	-
Leflunomide (LEF), n (%)	1080(12.18)	1080(23.17)	-	-

**Table 2:** Comparison of Baseline Characteristics Between RA and Non-RA Patients After Propensity Score Matching

Character	Total	RA Group	Non-RA Group	*P*-value	SMD
Basic Information	3104	1552	1552	-	-
Gender (Male), n (%)	2081(67.04)	1020(65.72)	1061(68.36)	0.137	0.026
Age (years)	65.37 ± 12.25	66.19 ± 12.35	64.55 ± 12.09	0.001	0.123
Lung Cancer, n (%)	55(1.77)	36(2.32)	19(1.22)	0.021	-
squamous cell carcinoma	32(1.03)	23(1.48)	9(0.58)	0.013	-
adenocarcinoma	15(0.48)	6(0.38)	9(0.58)	0.437	-
small cell carcinoma	3(0.09)	2(0.13)	1(0.06)	0.564	-
Others (Radiological Diagnosis)	5(0.16)	5(0.32)	0	0.025	-
Systolic BP (SBP, mmHg)	138.87 ± 20.34	139.80 ± 19.85	137.93 ± 20.78	0.012	0.104
Diastolic BP (DBP, mmHg)	85.95 ± 12.42	87.90 ± 12.65	83.99 ± 11.87	0.001	0.309
Hypertension, n (%)	1177(37.92)	508(32.73)	669(43.11)	0.001	0.112
Diabetes, n (%)	496(15.98)	245(15.79)	251(16.17)	0.769	0.001
Hyperlipidemia, n (%)	311(10.02)	179(11.53)	132(8.51)	0.005	0.030
Fasting Blood Glucose (mmol/L)	5.77 ± 2.03	5.55 ± 2.01	6.00 ± 2.02	0.001	0.213
Total Cholesterol (CHO, mmol/L)	4.42 ± 1.21	4.50 ± 1.20	4.34 ± 1.21	0.001	0.134
Triglycerides (TG, mmol/L)	1.57 ± 2.07	1.33 ± 2.50	1.84 ± 1.42	0.001	0.277
High-Density Lipoprotein (HDL, mmol/L)	1.04 ± 0.32	1.10 ± 0.35	0.99 ± 0.28	0.001	0.352
Low-Density Lipoprotein (LDL, mmol/L)	2.70 ± 0.93	2.81 ± 0.92	2.58 ± 0.92	0.001	0.244
Alanine Aminotransferase (ALT,U/L)	21.54 ± 22.15	19.08 ± 18.23	24.08 ± 25.33	0.001	0.209
Aspartate Aminotransferase (AST,U/L)	20.45 ± 14.28	19.47 ± 13.51	21.46 ± 14.98	0.001	0.120
C-Reactive Protein (CRP,mg/L)	30.84 ± 42.94	32.67 ± 42.09	27.66 ± 44.22	0.011	0.120
Erythrocyte Sedimentation Rate (ESR, mm/h)	45.26 ± 33.80	49.03 ± 33.44	38.83 ± 33.45	0.001	0.318
RA Duration (years)			-	-	-
Rheumatoid Factor (RF,IU/mL)	504(16.24)	504(32.47)	-	-	-
Cyclic Citrullinated Peptide (CCP,IU/mL)	344(11.08)	344(22.16)	-	-	-
Medication Use	680(21.91)	680(43.81)	-	-	-
Use of Lipid-Lowering Drugs, n (%)	220(7.09)	220(14.18)	-	-	-
Use of Antidiabetic Drugs, n (%)	301(9.70)	301(19.39)	-	-	-
Use of Antihypertensive Drugs, n (%)	12(0.39)	12(0.77)	-	-	-
Steroids, n (%)	85(2.74)	85(5.48)	-	-	-
Immunosuppressants, n (%)	34(1.10)	34(2.19)	-	-	-
Baricitinib, n (%)	273(8.80)	273(17.59)	-	-	-

### Univariable and multivariable analysis

In the matched cohort, we explored the association between RA and lung cancer risk through logistic regression analysis. Univariable analysis showed that RA was significantly associated with overall lung cancer risk (OR = 1.916, 95% CI: 1.094–3.355, *P* = 0.023). When we further conducted specialized analysis for squamous cell lung cancer, the association between RA and squamous cell carcinoma was particularly prominent (OR = 2.579, 95% CI: 1.189–5.591, *P* = 0.016). Additionally, multiple demographic, metabolic, and inflammatory indicators also showed associations (see Table 3 for details). In multivariable analysis, we adjusted for potential confounders including sex, age, hypertension, hyperlipidemia, and diabetes. After adjustment, the association between RA and overall lung cancer remained significant (adjusted OR = 1.850, 95% CI: 1.049–3.264, *P* = 0.034), and its strong association with squamous cell carcinoma remained robust after multivariable adjustment (adjusted OR = 2.415, 95% CI: 1.104–5.283, *P* = 0.027). Meanwhile, multiple variables including blood pressure, lipids, glucose, and inflammatory markers (ESR, CRP) remained independent correlates of RA after adjustment (Table 3).

**Table 3:** Logistic Regression Analysis

Characteristic	Univariable		Multivariable	
	OR(95%CI)	P	OR(95%CI)	P
Age (years)	1.011 (1.005 ~ 1.017)	0.001	**-**	
Gender (Male)	0.887 (0.764 ~ 1.031)	0.118	-	
Hypertension	0.642 (0.555 ~ 0.743)	0.001	-	
Diabetes	0.972 (0.802 ~ 1.177)	0.769	-	
Hyperlipidemia	1.402 (1.107 ~ 1.777)	0.005	-	
Lung Cancer	1.916 (1.094 ~ 3.355)	0.023	**1.850 (1.049 ~ 3.264)**	**0.034**
squamous cell carcinoma	2.579 (1.189 ~ 5.591)	0.016	**2.415 (1.104 ~ 5.283)**	**0.027**
SBP (mmol/L)	1.005 (1.001 ~ 1.008)	0.012	1.006 (1.002 ~ 1.010)	0.001
DBP (mmol/L)	1.027 (1.020 ~ 1.033)	0.001	1.029 (1.022 ~ 1.035)	0.001
ALT (U/L)	0.987 (0.983 ~ 0.991)	0.001	0.987 (0.983 ~ 0.992)	0.001
AST(U/L)	0.990 (0.984 ~ 0.995)	0.001	0.990 (0.984 ~ 0.995)	0.001
CHO (mmol/L)	1.115 (1.049 ~ 1.186)	0.001	1.103 (1.033 ~ 1.178)	0.004
TG (mmol/L)	0.663 (0.608 ~ 0.722)	0.001	0.652 (0.595 ~ 0.713)	0.001
HDL (mmol/L)	2.883 (2.257 ~ 3.682)	0.001	2.773 (2.142 ~ 3.589)	0.001
LDL (mmol/L)	1.305 (1.203 ~ 1.416)	0.001	1.275 (1.172 ~ 1.387)	0.001
Blood Glucose (mmol/L)	0.884 (0.848 ~ 0.922)	0.001	0.881 (0.843 ~ 0.920)	0.001
ESR(mm/h)	1.009 (1.007 ~ 1.012)	0.001	1.009 (1.007 ~ 1.012)	0.001
CRP (mg/L)	1.003 (1.001 ~ 1.005)	0.011	1.002 (1.000 ~ 1.005)	0.036

### Subgroup analysis of squamous cell lung cancer and RA stratified by different factors

Subgroup analysis showed that the OR for squamous cell carcinoma in the age < 60 years subgroup was 0.93 (95%CI: 0.86–1.01, *P* = 0.098), while in the age ≥ 60 years subgroup the OR was 1.00 (95%CI: 0.96–1.04, *P* = 0.991). Sex subgroup analysis showed that the OR for squamous cell lung cancer in females was 0.98 (95%CI: 0.92–1.04, *P* = 0.483), and in males was 0.99 (95%CI: 0.95–1.05, *P* = 0.982). The association in the ESR < 45 group (OR = 1.00, 95%CI: 0.95–1.05, *P* = 0.982) showed no significant difference from the ESR ≥ 45 group (OR = 0.98, 95%CI: 0.94–1.03, *P* = 0.475). The association in the CRP < 30 group (OR = 1.00, 95%CI: 0.95–1.05, *P* = 0.881) was similarly no different from the CRP ≥ 30 group (OR = 0.97, 95%CI: 0.93–1.02, *P* = 0.285). These subgroup analysis results indicate that the association between squamous cell lung cancer and RA is relatively stable across different subgroups (stratified by age, sex, ESR, and CRP levels), with no significant effect modification observed. Detailed results are shown in Table 4.

**Table 4:** Subgroup Analysis of Squamous Cell Lung Cancer and RA

Component	OR (95%CI) Age < 60 (*n* = 879)	OR (95%CI) Age ≥ 60 (*n* = 2225)	OR (95%CI) Female (*n* = 1023)	OR (95%CI) Male (*n* = 2081)	OR (95%CI) ESR < 45 (*n* = 1182)	OR (95%CI) ESR ≥ 45 (*n* = 905)	OR (95%CI) CRP < 30 (*n* = 1428)	OR (95%CI) CRP ≥ 30 (*n* = 634)
squamous cell carcinoma	0.93 (0.86, 1.01) (*P* = 0.098)	1.00 (0.96, 1.04) (*P* = 0.991)	9.98 (0.92, 1.04) (*P* = 0.483)	0.99 (0.95, 1.03) (*P* = 0.558)	1.00 (0.95, 1.05) (*P* = 0.982)	0.98 (0.94, 1.03) (*P* = 0.475)	1.00 (0.95, 1.05) (*P* = 0.881)	0.97 (0.93, 1.02) (*P* = 0.285)
SBP (mmHg)	1.04 (0.96, 1.13) (*P* = 0.285)	1.0 (0.97, 1.02) (*P* = 0.988)	0.99 (0.95, 1.02) (*P* = 0.548)	1.02 (0.98, 1.05) (*P* = 0.307)	1.02 (0.96, 1.08) (*P* = 0.461)	1.0 (0.97, 1.03) (*P* = 0.946)	1.0 (0.96, 1.05) (*P* = 0.87)	1.01 (0.98, 1.05) (*P* = 0.452)
DBP (mmHg)	0.98 (0.87, 1.10) (*P* = 0.789)	1.01 (0.97, 1.04) (*P* = 0.804)	1.02 (0.96, 1.09) (*P* = 0.421)	0.99 (0.94, 1.04) (*P* = 0.572)	1.01 (0.93, 1.09) (*P* = 0.857)	0.99 (0.94, 1.05) (*P* = 0.808)	1.01 (0.94, 1.08) (*P* = 0.816)	0.99 (0.94, 1.04) (*P* = 0.768)
ALT (U/L)	0.98 (0.87, 1.03) (*P* = 0.67)	0.96 (0.89, 1.01) (*P* = 0.166)	0.93 (0.81, 1.02) (*P* = 0.172)	0.97 (0.91, 1.02) (*P* = 0.366)	0.96 (0.79, 1.08) (*P* = 0.566)	0.94 (0.85, 1.01) (*P* = 0.181)	0.88 (0.76, 0.99) (*P* = 0.062)	0.98 (0.91, 1.02) (*P* = 0.4)
AST (U/L)	1.05 (0.93, 1.13) (*P* = 0.269)	1.03 (0.96, 1.07) (*P* = 0.304)	1.04 (0.93, 1.10) (*P* = 0.275)	1.02 (0.96, 1.07) (*P* = 0.392)	1.0 (0.82, 1.08) (*P* = 1.0)	1.03 (0.95, 1.09) (*P* = 0.329)	1.06 (0.97, 1.12) (*P* = 0.115)	1.02 (0.96, 1.07) (*P* = 0.357)
CHO (mmol/L)	0.52 (0.01, 12.53) (*P* = 0.715)	0.87 (0.33, 2.55) (*P* = 0.812)	0.42 (0.10, 2.21) (*P* = 0.265)	2.32 (0.59, 13.09) (*P* = 0.291)	0.27 (0.09, 3.93) (*P* = 0.474)	0.91 (0.33, 2.89) (*P* = 0.857)	2.55 (0.26, 20.91) (*P* = 0.372)	0.54 (0.08, 1.59) (*P* = 0.345)
TG (mmol/L)	1.16 (0.14, 4.67) (*P* = 0.825)	0.9 (0.37, 1.06) (*P* = 0.793)	1.39 (0.48, 2.75) (*P* = 0.432)	0.54 (0.16, 1.01) (*P* = 0.253)	0.72 (0.03, 1.03) (*P* = 0.814)	1.18 (0.48, 2.05) (*P* = 0.63)	0.29 (0.03, 0.96) (*P* = 0.243)	1.75 (0.77, 3.55) (*P* = 0.116)
HDL (mmol/L)	0.07 (0.00, 10.80) (*P* = 0.35)	2.98 (0.40, 15.35) (*P* = 0.258)	1.91 (0.09, 26.81) (*P* = 0.653)	1.28 (0.08, 10.63) (*P* = 0.839)	18.82 (0.29, 77.99) (*P* = 0.226)	2.76 (0.31, 18.79) (*P* = 0.319)	2.56 (0.14, 53.23) (*P* = 0.508)	2.41 (0.26, 23.86) (*P* = 0.42)
LDL (mmol/L)	2.23 (0.06, 169.33) (*P* = 0.684)	1.35 (0.48, 3.41) (*P* = 0.597)	2.55 (0.46, 10.08) (*P* = 0.208)	0.56 (0.10, 2.20) (*P* = 0.482)	1.42 (0.10, 59.70) (*P* = 0.848)	1.4 (0.42, 3.79) (*P* = 0.569)	0.33 (0.04, 3.27) (*P* = 0.309)	2.78 (0.80, 22.59) (*P* = 0.187)
Blood Glucose (mmol/L)	1.12 (0.64, 1.36) (*P* = 0.358)	1.01 (0.76, 1.22) (*P* = 0.951)	0.91 (0.52, 1.25) (*P* = 0.654)	1.08 (0.85, 1.22) (*P* = 0.347)	0.84 (0.66, 1.25) (*P* = 0.668)	1.06 (0.82, 1.25) (*P* = 0.597)	0.97 (0.56, 1.22) (*P* = 0.895)	0.99 (0.75, 1.21) (*P* = 0.944)

### Nonlinear relationships between clinical parameters and squamous cell lung cancer

**Fig. 4:**
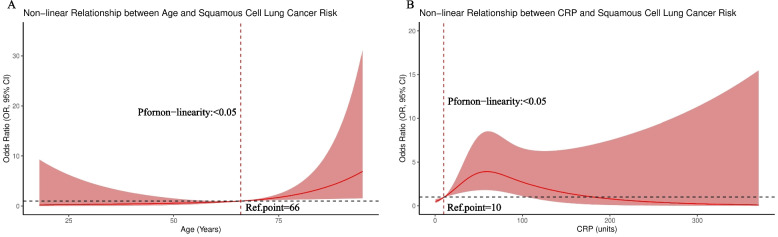
Nonlinear Relationships Between Clinical Parameters and Squamous Cell Lung Cancer Risk

Restricted cubic spline model analysis revealed significant nonlinear relationships between clinical parameters and squamous cell lung cancer (Fig. 4). Regarding the relationship between age and squamous cell lung cancer, age showed a pattern of initial stability followed by increase (*P* < 0.05), with risk reaching the reference point (OR = 1.0) at approximately 66 years, after which risk gradually increased. Specifically, in the 40–66 year range, the relationship between age and squamous cell lung cancer risk was relatively stable, maintaining a lower risk level; when age exceeded 66 years, squamous cell lung cancer risk showed a significant upward trend, reaching a higher level (OR > 2.0) above 80 years. C-reactive protein (CRP) and squamous cell lung cancer risk showed a bell-shaped relationship (*P* < 0.05), with reference point at 10. At low CRP levels (< 10 mg/L), risk showed a slight upward trend; when CRP levels were in the 10–50 mg/L range, risk increased significantly, reaching a peak (OR > 2.5) around 20–30 mg/L; however, when CRP levels exceeded 50 mg/L, risk began to decline, falling below baseline level (OR < 1.0) at 200 mg/L. The biological mechanisms of this nonlinear relationship are unclear and may involve multiple factors, including different CRP levels possibly reflecting different pathophysiological states (such as acute infection, other serious diseases, etc.). This observation requires further validation and interpretation in future studies, particularly considering the effects of disease activity, comorbidities, and treatment on CRP levels.

### Performance evaluation and comparison of prediction models

**Fig. 5:**
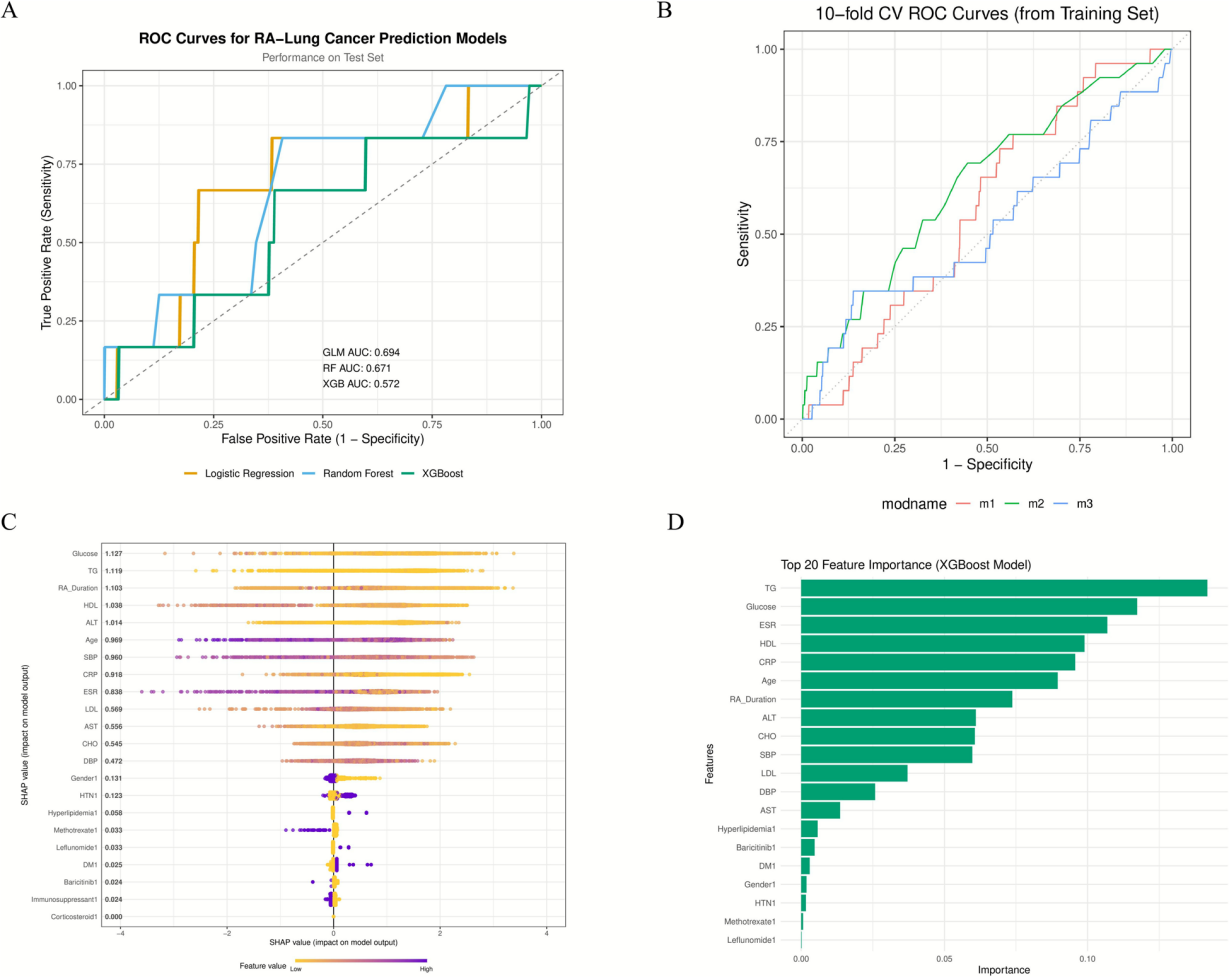
Performance Evaluation of Machine Learning Models for Predicting Squamous Cell Lung Cancer Risk in RA Patients Based on Clinical Features. **A** Test set ROC curves. Shows ROC curves and AUC values for logistic regression (GLM), random forest (RF), and XGBoost (XGB). Gray dashed line represents random guessing level (AUC = 0.5). **B** tenfold cross-validation ROC curves. Shows average ROC curves and standard deviation ranges for three models in cross-validation. **C** SHAP value analysis. Shows the contribution of each feature to model output. Color indicates feature value level (red = high, blue = low). **D** XGBoost model feature importance ranking (Top 20). TG, blood glucose, ESR, HDL, and CRP are the most important predictors

Given the significant association between RA and squamous cell lung cancer (OR = 2.415, *P* = 0.027), we constructed machine learning models based on 22 clinical features. Test set AUCs were: logistic regression 0.684, random forest 0.671, XGBoost 0.572 (Fig. 5A). Ten-fold cross-validation showed average AUC of 0.64–0.73, standard deviation 0.12–0.18 (Fig. 5B). Feature importance analysis (Fig. 5C) showed TG, blood glucose, ESR, HDL, and CRP contributed most. SHAP analysis (Fig. 5D) showed high values of TG, blood glucose, and inflammatory indicators were associated with increased risk.

### Mendelian randomization analysis (European Population) reveals preliminary paradox: apparent protective effect of ra on squamous cell lung cancer

**Fig. 6:**
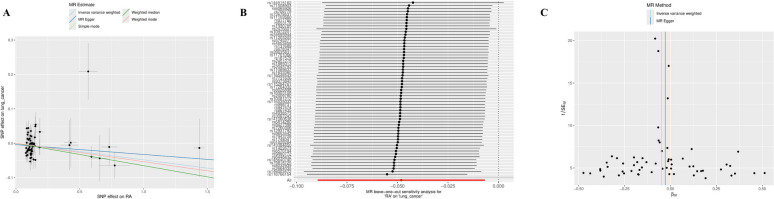
Mendelian Randomization Sensitivity Analysis of RA on Squamous Cell Lung Cancer Risk. **A** Scatter plot showing the effect values of each instrumental variable on RA (X-axis) and squamous cell lung cancer (Y-axis). The slopes in the figure represent the overall causal effects estimated by different MR methods. **B** Leave-one-out sensitivity analysis plot showing the impact of remaining variables on overall causal effect after removing each instrumental variable one by one. **C** Funnel plot for assessing heterogeneity of instrumental variables

To explore the causal relationship between RA and squamous cell lung cancer from a genetic perspective, we turned to use completely independent European population GWAS data, first conducting univariable Mendelian randomization (UVMR) analysis. It should be emphasized that the population used in this genetic analysis (European ancestry) is completely different from our clinical cohort (Chinese population). Unexpectedly, the inverse variance weighted (IVW) method results, as the main analysis method, showed that genetically predicted RA had a significant protective effect on squamous cell lung cancer (OR = 0.985, 95% CI: 0.974–0.996, *P* = 0.016). This negative association was consistently supported by multiple sensitivity analysis methods, for example, the weighted median method also yielded results with the same direction and statistical significance (OR = 0.980, *P* = 0.012; Table 5). A series of rigorous tests further confirmed the robustness of this result. We found no significant heterogeneity among instrumental variables (Cochran's Q *P* > 0.8), horizontal pleiotropy (MR-Egger intercept *P* = 0.781; MR-PRESSO *P* = 0.88), or single SNP-driven effects (leave-one-out analysis), indicating that this protective association was not caused by common statistical biases (Fig. 6). This preliminary genetic analysis result formed a sharp contrast with our clinical observations (RA increases squamous cell lung cancer risk), suggesting that important confounding factors may exist that distort the estimation of genetic associations.

**Table 5:** Mendelian Randomization Analysis Results of RA on Squamous Cell Lung Cancer Risk

Exposure	Outcome	nSNP	Method	Beta	*P*-value	OR	95% CI
RA	Squamous Cell Lung Cancer	60	MR Egger	−0.0286	0.4148	0.9718	(0.9078, 1.0404)
RA	Squamous Cell Lung Cancer	60	Weighted Median	−0.0651	0.0263	0.937	(0.8847, 0.9924)
RA	Squamous Cell Lung Cancer	60	Inverse Variance Weighted	−0.0481	0.0227	0.953	(0.9144, 0.9933)
RA	Squamous Cell Lung Cancer	60	Simple Mode	−0.0535	0.3092	0.9479	(0.8559, 1.0499)
RA	Squamous Cell Lung Cancer	60	Weighted Mode	−0.0535	0.064	0.9479	(0.8968, 1.0021)

### Genetic correlation analysis between RA and squamous cell lung cancer

To assess the degree of genetic sharing between rheumatoid arthritis and squamous cell lung cancer at the genome-wide level, we performed linkage disequilibrium score regression (LDSC) analysis. As shown in Table 6, we first estimated the SNP heritability (h2snp) of both diseases. Analysis showed that both RA (h2snp = 0.109, SE = 0.014, *P* = 3.36 × 10⁻⁹) and squamous cell lung cancer (h2snp = 0.058, SE = 0.012, *P* = 1.58 × 10⁻⁶) showed statistically significant heritability, indicating that common genetic variants play important roles in the development of both diseases. However, the core genetic correlation analysis results indicated that there was no significant genetic correlation between RA and squamous cell lung cancer (rg = −0.019, SE = 0.078, *P* = 0.806). This result suggests that although the two diseases are clinically associated, their occurrence is not driven by widely shared common genetic backgrounds, and their pathogenic genetic factors are largely independent. This result indicates that although the two diseases are clinically associated, they do not share extensive genetic foundations at the genome-wide level. This finding, together with the preliminary UVMR analysis results, forms an interesting paradox—although the two diseases do not have significant widespread genetic associations, specific RA genetic instrumental variables show a protective effect on squamous cell lung cancer.

**Table 6:** LDSC Analysis Results of SNP Heritability and Genetic Correlation Between RA and Squamous Cell Lung Cancer

Exposure (Trait 1)	Outcome (Trait 2)	Genetic Correlation (rg)	Standard Error (se)	*P*-value	Heritability of RA (h_snp2)	Heritability of Squamous Cell Lung Cancer (h_snp2)
Rheumatoid Arthritis	Squamous Cell Lung Cancer	−0.019	0.078	0.806	0.109 (SE = 0.014)	0.058 (SE = 0.012)

### Multivariable mendelian randomization (MVMR) analysis: resolving the paradox and establishing causality

To resolve the "protective effect" paradox in univariable MR analysis and explore the confounding role of smoking in the RA-squamous cell lung cancer relationship, we further implemented multivariable Mendelian randomization (MVMR) analysis. Analysis results showed that after adjusting for genetic predisposition to smoking, the causal relationship between RA and squamous cell lung cancer underwent a critical reversal. Results based on the inverse variance weighted (IVW) method indicated that genetically predicted RA significantly increased squamous cell lung cancer risk (OR = 1.02, 95% CI: 1.00–1.03, *P* = 0.046). This finding was consistently confirmed in MR-Egger regression (OR = 1.02, *P* = 0.046), successfully resolving the UVMR paradox and achieving consistency with our clinical observation results. Sensitivity analyses supported the robustness of this result. The MR-Egger regression intercept term was not significant (*P* = 0.543), indicating no obvious horizontal pleiotropy was found. However, Cochran's Q test revealed significant heterogeneity among instrumental variables (*P* < 0.001), suggesting this factor should be considered when interpreting results. Notably, after adjusting for genetic effects of RA in the model, the causal association between smoking and squamous cell lung cancer was no longer statistically significant (*P* > 0.05). Detailed results are shown in Table 7. This MVMR analysis successfully resolved the contradiction we observed earlier, establishing the causal risk effect of RA on squamous cell lung cancer in European populations. It should be emphasized again that this genetic causal conclusion comes from European populations and cannot be directly used to explain the clinical associations observed in our Chinese cohort. Most importantly, this analysis highlights the role of smoking as a key confounding factor, which may mask the true relationship between RA and squamous cell lung cancer in unadjusted analyses.

**Table 7:** Multivariable mendelian randomization and sensitivity analysis of rheumatoid arthritis and smoking on squamous cell lung cancer risk

Outcome	Exposure	nsnp	Method	OR (95% CI)	*p* value	Cochran’s Qp
Squamous Cell Lung Cancer	RA	2198	Inverse variance weighted	1.02 (1.00, 1.03)	0.046	< 0.001
			MR-Egger	1.02 (1.00, 1.03)	0.046	
			Median method	1.00 (0.98, 1.03)	0.842	
	Smoking	2198	Inverse variance weighted	1.03 (0.94, 1.14)	0.516	
			MR-Egger	1.00 (0.88, 1.15)	0.954	
			Median method	1.08 (0.94, 1.24)	0.265	

### Identification of shared causal genes between RA and squamous cell lung cancer

**Fig. 7:**
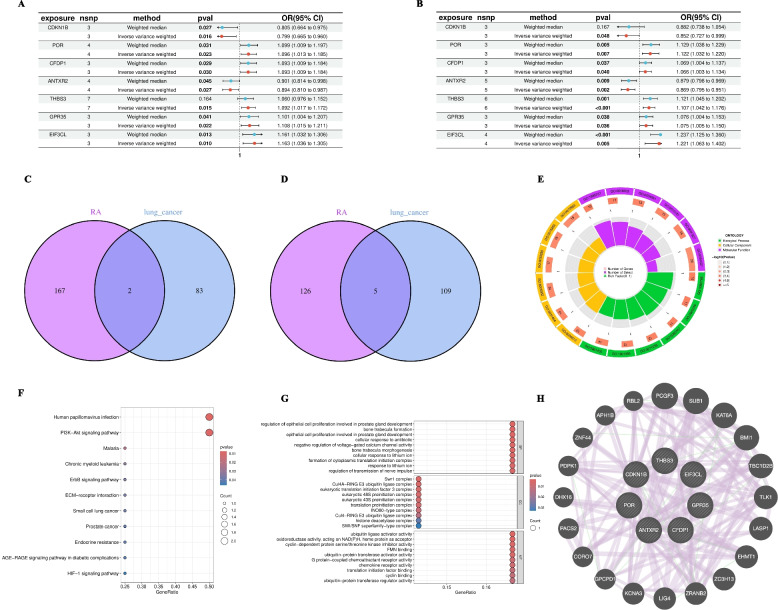
Identification of Shared Causal Genes Between RA and Squamous Cell Lung Cancer and Their Functional Analysis (Exploratory Analysis). **A**, **B** Forest plots of Mendelian randomization analysis of 7 shared genes with RA risk (**A**) and squamous cell lung cancer risk (**B**). OR > 1 indicates risk factor, OR < 1 indicates protective factor. **C**, **D** Venn diagrams identifying shared protective genes (**C**) and risk genes (**D**) between RA and squamous cell lung cancer. Figure C shows 2 shared protective genes, Figure D shows 5 shared risk genes. **E** GO functional enrichment analysis circular diagram of shared genes, showing enrichment results at three levels: biological processes, cellular components, and molecular functions. **F** KEGG pathway enrichment analysis bubble plot of shared genes. Bubble color represents *P*-value significance, bubble size represents number of enriched genes. **G** Detailed item diagram of GO functional enrichment analysis, showing specific enrichment items and their significance. **H** PPI network diagram of 7 shared genes and their interacting proteins, highlighting CDKN1B and POR as potential key hub nodes. Note: Shared genes were identified through single-gene Mendelian randomization analysis (*P* < 0.05), functional enrichment analysis provides preliminary evidence of potential shared mechanisms

After confirming the causal risk effect of RA on squamous cell lung cancer through MVMR (in European population), we further explored potential shared molecular mechanisms behind it. It should be specifically noted that the following analysis used more lenient thresholds (P < 0.05 for single-gene MR) and has a limited number of genes, should be regarded as highly exploratory findings. Through conducting single-gene MR analysis separately for both diseases and taking the intersection of results, we successfully identified 7 shared causal genes. As shown in forest plots (Figs. 7A, B), these 7 genes show highly consistent causal effect directions on RA and squamous cell lung cancer risk (for example, POR and EIF3CL are risk factors for both diseases, while ANTXR2 is a protective factor for both), indicating they are key genetic nodes connecting the pathophysiological processes of both diseases. The identification process of these genes is shown through Venn diagrams (Figs. 7C, D): we ultimately identified 5 common risk genes (POR, CFDP1, THBS3, GPR35, EIF3CL) and 2 common protective genes (CDKN1B, ANTXR2). To explore the biological functions of these shared genes, we conducted exploratory functional enrichment analysis. GO functional enrichment analysis circular diagram (Fig. 7E) shows enrichment results at three levels: biological processes, cellular components, and molecular functions. KEGG pathway enrichment analysis bubble plot (Fig. 7F) suggests that shared genes may be involved in multiple pathways including PI3K-Akt signaling pathway. However, among 7 shared genes, only 2 genes map to the PI3K-Akt pathway. Given the limited number of genes mapping to this pathway, current results can only suggest that the PI3K-Akt pathway may be related, and its exact role in the association between the two diseases still requires larger-scale studies and experimental validation. GO functional enrichment analysis detailed item diagram (Fig. 7G) further reveals specific molecular functions such as "eukaryotic translation initiation factor 3 complex" and "ubiquitin-protein transferase activator activity". Finally, to elucidate the interaction relationships among these genes, we constructed a protein–protein interaction (PPI) network (Fig. 7H). Network analysis shows that CDKN1B, as a key cell cycle regulator, and POR, as an oxidoreductase, are key hub nodes (Hub genes) in the network, having close interactions with other proteins in the network.

## Discussion

This study adopted an evidence triangulation strategy, providing independent evidence in two different populations: Chinese clinical cohort shows RA is significantly associated with squamous cell lung cancer (but not other subtypes) risk (OR = 2.415), European population genetic analysis supports RA as a causal risk factor for squamous cell lung cancer (OR = 1.02, *P* = 0.046). It should be emphasized that these two pieces of evidence come from different populations (China vs Europe) and different study designs (observational vs genetic causation), and should be regarded as mutually complementary rather than mutually validating independent findings.

The core finding of this study is the significant subtype specificity of the RA-lung cancer association. Detailed subtype analysis shows that RA significantly increases squamous cell lung cancer risk (adjusted OR = 2.415, 95%CI: 1.104–5.283, *P* = 0.027), but shows no significant association with adenocarcinoma (0.38% vs. 0.58%, P = 0.437) and small cell carcinoma (0.13% vs. 0.06%, *P* = 0.564). This finding is significant: First, it suggests that RA-related chronic inflammation, autoantibodies, or therapeutic drugs may specifically promote squamous cell carcinoma development. Lung adenocarcinoma originates from peripheral airway glandular cells, while squamous cell carcinoma originates from central airway squamous epithelium, with significantly different cellular origins, driver mutation spectra, and microenvironment requirements [22, 23]. Second, this helps explain inconsistent results in previous literature on RA-lung cancer associations, as most studies treating lung cancer as a single endpoint may dilute specific signals. Third, this suggests screening strategies for RA patients should be optimized for squamous cell lung cancer rather than adopting the same strategy for all lung cancer types.

In genetic analysis, we discovered and resolved an important paradox. Preliminary univariable Mendelian randomization (UVMR) unexpectedly showed RA had a protective effect on squamous cell lung cancer (OR = 0.985), contrasting with clinical observations. Linkage disequilibrium score regression (LDSC) further showed no significant genetic correlation between the two diseases (rg = −0.019, *P* = 0.806). Through multivariable Mendelian randomization (MVMR) after adjusting for genetic effects of smoking, we successfully resolved this contradiction, indicating that RA indeed increases squamous cell lung cancer risk (OR = 1.02, *P* = 0.046). This shift from "apparent protection" to "confirmed risk" reveals the profound role of smoking as a key confounding factor and demonstrates the importance of methodological innovation in complex disease association research. Our findings echo the work of Wu et al. [3], emphasizing the necessity of considering key confounders in genetic causal inference. Notably, the clinical effect size (OR = 2.415) is significantly larger than the genetic effect (OR = 1.02), which is mainly attributed to population differences (China vs Europe) and study design differences (observational vs genetic causation). Other possible factors include genetic instruments capturing lifetime exposure effects while clinical diagnosis reflects short-term risk, and the influence of residual confounding [24], but these explanations need validation in future studies.

Large-scale propensity score matching cohort study shows RA is strongly associated with squamous cell lung cancer (OR = 2.415), and this association remains stable across subgroups of different ages, genders, and comorbidity statuses. Nonlinear relationship analysis revealed that age (particularly > 66 years) and moderate inflammatory status (CRP 20–30 mg/L) are key features of increased squamous cell lung cancer risk, providing reference for identifying high-risk RA patients. However, due to retrospective design limitations, we were unable to collect disease activity scores (such as DAS28) and the effects of immunosuppressive treatment on inflammation control, therefore cannot determine whether CRP-squamous cell lung cancer association can be improved through aggressive anti-inflammatory treatment [25].

When exploring machine learning prediction feasibility, all models (logistic regression, random forest, and XGBoost) showed limited predictive ability (AUC = 0.57–0.68). This reveals the inherent challenges of individual risk prediction through routine clinical parameters, reflecting the "predictability paradox" between population effects and individual prediction. Although clinical analysis shows significant population-level association (OR = 2.415), statistical associations are difficult to translate into individual predictive ability. Notably, metabolic abnormalities (TG, blood glucose) and systemic inflammation (ESR, CRP) are the most important predictive factors, which is consistent with clinical association analysis, providing clues for understanding the biological mechanisms of RA-squamous cell lung cancer association. Model performance limitations are attributed to sample size restrictions (32 cases of squamous cell lung cancer), missing key variables (smoking history, genetic markers), and complex gene-environment interactions, with all models based on single-center data and lacking external validation.

As an exploratory analysis, we identified seven genes with consistent causal effects on both diseases. Functional enrichment analysis suggests they may be involved in multiple biological pathways, including the PI3K-Akt signaling pathway [26]. Given this pathway's role in regulating cell survival, proliferation, and metabolism [27], this connection warrants future experimental validation, but current genetic evidence (only 2 genes in the PI3K-Akt pathway) is insufficient and should be interpreted cautiously. Among shared risk genes, THBS3 (thrombospondin 3) and EIF3CL (eukaryotic translation initiation factor 3 subunit C-like) show particularly strong associations with both diseases, possibly representing key molecular nodes connecting RA pathophysiology with squamous cell lung cancer development.

Our study has several significant strengths. In clinical cohort analysis, propensity score matching effectively balanced baseline characteristics, and subtype-specific analysis revealed the novel finding that RA is mainly associated with squamous cell lung cancer. In genetic analysis, MVMR represents methodological progress in addressing complex confounding relationships. However, we need to consider several important limitations. First, the most important limitation lies in cross-population inference issues. The clinical cohort (Chinese population) and Mendelian randomization analysis (European population) provide two separate, population-specific pieces of evidence, and genetic causal relationships found in European populations cannot be directly used to explain or confirm clinical associations observed in the Chinese cohort. East Asian and European populations may differ in genetic architecture, linkage disequilibrium patterns, allele frequencies, and gene-environment interactions [28], making dedicated MR studies in East Asian populations urgently needed for future validation. Second, in the observational cohort study, although propensity score matching and multivariable adjustment were performed, residual confounding from unmeasured factors cannot be completely ruled out, most importantly smoking data were not systematically collected in the retrospective records, and other unmeasured factors such as disease activity scores, comorbidities, and dynamic effects of treatment on inflammation control may also contribute to residual confounding.Retrospective design also cannot assess protective effects of treatment on squamous cell lung cancer risk. Third, genetic analysis also has shortcomings. Although GWAS data come from different sources, sample overlap between European population cohorts cannot be completely ruled out, which may slightly affect MR estimation precision, although LDSC analysis is insensitive to this. Meanwhile, although MVMR controlled for genetic predisposition to smoking, it cannot consider all aspects of smoking behavior and environmental tobacco exposure. Finally, the shared mechanism analysis must be regarded as highly exploratory, with two compounded methodological weaknesses: (1) single-gene MR used a lenient P < 0.05 threshold, facing high false-positive risk; (2) functional enrichment analysis using an extremely limited number (N = 7) of genes has severely insufficient statistical power. Therefore, all findings from this module (including the PI3K-Akt pathway) do not have robust inference value and can only serve as preliminary hypotheses for future research.

Despite these limitations, this study still has important clinical significance. RA patients, particularly those of advanced age (> 66 years) or with moderate inflammatory status, may need stricter squamous cell lung cancer screening strategies. Exploratory analysis of shared genes provides direction for future research but requires larger-scale genetic studies and experimental validation. The limitations of machine learning models in individual risk prediction highlight the necessity of developing more precise predictive tools, which may need to integrate genetic markers, detailed environmental exposure history, and advanced imaging features.

In summary, through evidence triangulation strategy, this study provides two levels of independent evidence. First, clinical cohort study in Chinese population shows RA is significantly associated with squamous cell lung cancer (but not other lung cancer subtypes) risk (OR = 2.415), and this subtype-specific finding is a key contribution of this study. Second, independent genetic causal inference (MVMR) in European population also supports RA as a causal risk factor for squamous cell lung cancer (OR = 1.02). It should be emphasized that these two levels of evidence come from different populations and methodologies and should be regarded as mutually complementary rather than mutually validating, and future MR validation in East Asian populations is urgently needed. The "protective effect" paradox shown by univariable MR was resolved through MVMR adjustment for smoking, ultimately establishing the causal risk effect of RA on squamous cell lung cancer. Clinical prediction models showed limited performance (AUC = 0.57–0.68), but the importance of metabolic and inflammatory markers provides clues for mechanistic research. Preliminary exploration of shared genetic pathways provides hypotheses for the biological basis of this association and requires further experimental validation. These findings may have important clinical implications: RA patients, particularly those of advanced age (> 66 years) or with moderate inflammatory status, may need enhanced screening for squamous cell lung cancer.

## Supplementary Information

Supplementary Material 1

Supplementary Material 2

## Declarations

### Data availability

Data are available from the corresponding author upon reasonable request.
